# Prenatal finding of isolated ventricular septal defect: genetic association, outcomes and counseling

**DOI:** 10.3389/fgene.2024.1447216

**Published:** 2024-10-02

**Authors:** Xin Chen, Qian Zhang, Man Lu, Qiuxia Feng, Litao Qin, Shixiu Liao

**Affiliations:** ^1^ Medical Genetics Institute of Henan Province, Henan Provincial Key Laboratory of Genetic Diseases and Functional Genomics, Henan Provincial People’s Hospital, People’s Hospital of Zhengzhou University, Zhengzhou University, Zhengzhou, China; ^2^ National Health Commission Key Laboratory of Birth Defects Prevention, Zhengzhou, China; ^3^ Reproduction Medical Center, Henan Provincial People’s Hospital, Zhengzhou University People’s Hospital, Zhengzhou, China

**Keywords:** isolated ventricular septal defect, prenatal diagnosis, genetic counseling, pregnancy outcome, molecular genetic technologies

## Abstract

The innovation in ultrasound has greatly promoted the prenatal diagnosis of ventricular septal defect. As a minor lesion of congenital heart disease, the prenatal genetic counseling of isolated ventricular septal defect faces some challenges, including the true genetic correlationship, selection of appropriated testing methods to identify deleterious mutations, and avoidance of overdiagnosis and overintervention. Researchers have explored the prenatal diagnosis efficiency of commonly used cytogenetic and molecular genetic technologies. Small insertions/deletions and monogenic variants with phenotypic heterogeneity play important role and contribute to the comprehend of pathogenesis. Isolated ventricular septal defect fetuses without genetic finding and extracardiac structural abnormality generally have good pregnancy outcome. Long-term follow-up data is needed to describe the comprehensive map, such as the potential missed diagnosis especially late-onset syndromes, the impact on the quality of life and life expectancy. When conducting prenatal genetic counseling, strict adherence to ethical principles is needed to ensure that the rights of all parties involved are fully protected. Clinicians should carefully evaluate the risks and benefits and provide parents with sufficient information and advice to enable them to make informed decisions.

## 1 Introduction

Congenital heart disease (CHD) is the most common birth defect and leading cause of global burden of disease (Authoranonmys, 2020; Roth et al., 2020). As the most frequent type of CHD, ventricular septal defect (VSD) has intricate etiology and Pathogenesis (Nora, 1968; Goor et al., 1970; Pierpont et al., 2007). Sonography is the primary imaging modality for diagnosing and monitoring VSDs. Over the past 30 years, the development and growth of fetal echocardiography has been driven by technical innovation (Rajiah et al., 2011; Maulik et al., 2017). The resolution improvement and operation standardization have pushed the boundaries of what can be seen and measured, and helped the advance period of diagnosis for small VSDs to fetal stage, resulted in the intense demand for prenatal counseling (van der Linde et al., 2011; Yagel et al., 2011; Boehme et al., 2022; Dawood et al., 2022). Genetics plays an important role in the pathogenesis of VSD, and this discovery benefits from the great technological progress in human genome research. The aim of this review is to explain the genetic association and clinical outcomes for fetuses with isolated VSD, so as to provide comprehensive basis for prenatal genetic counseling.

## 2 Search strategy and selection criteria

Articles referenced in this manuscript were identified by MEDLINE and EMBASE. The last electronic search was performed on 13 September 2024 utilizing combinations of the relevant medical subject heading (MeSH) terms, word variants, and keywords for “fetus, prenatal, isolated and ventricular septal defect”. The bibliography of high-impact articles were reviewed to identify additional relevant studies and was included in our references when appropriate.

**FIGURE 1:**
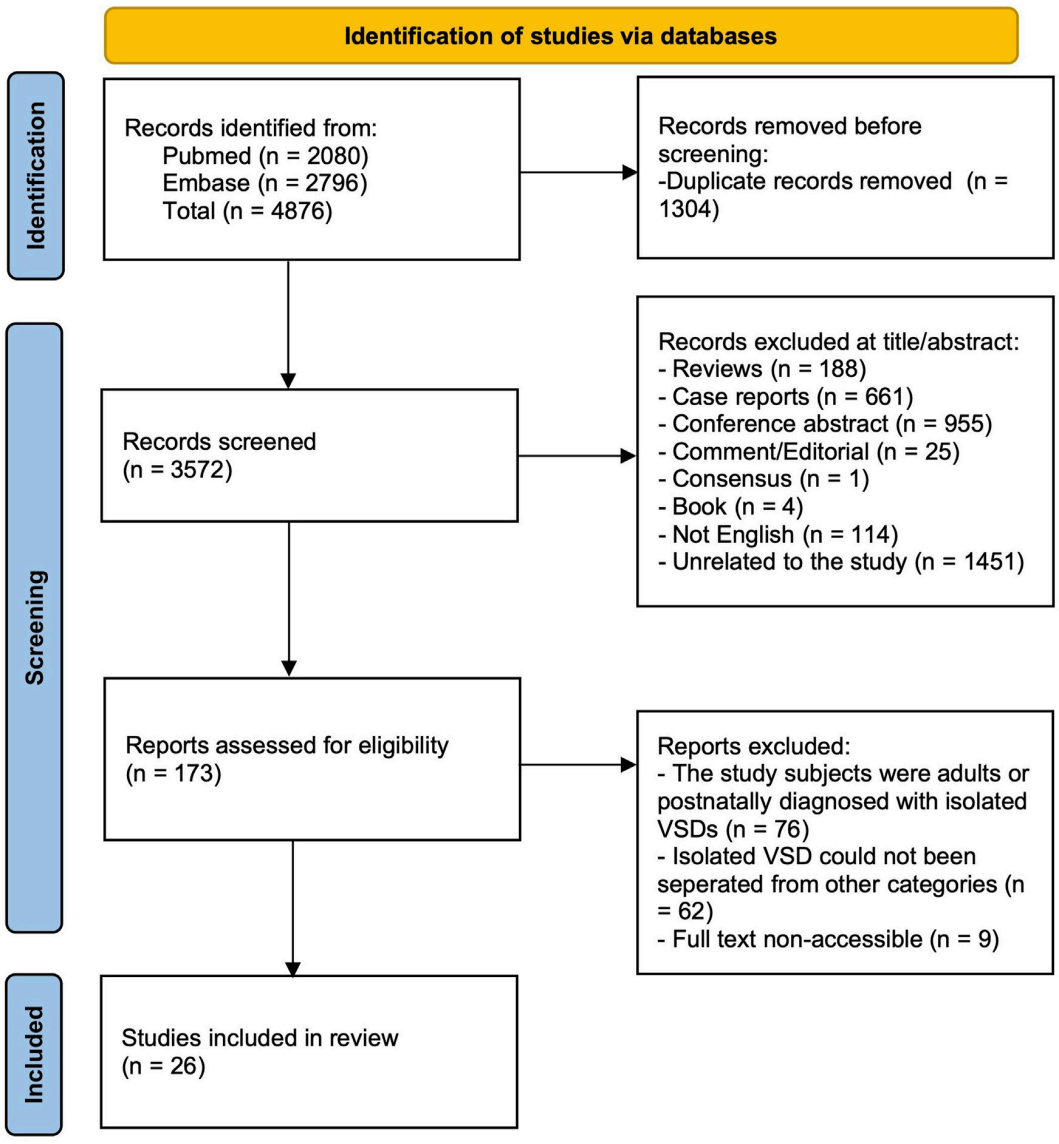
Flow chart of included articles to evaluate the risk of genetic abnormalities and clinical outcomes of fetuses with isolated VSD.

Additional articles were included to evaluate the risk of genetic abnormalities and clinical outcomes of fetuses with isolated VSD if the following criteria were met (Figure 1): (1) fetuses were diagnosed as isolated VSD by prenatal ultrasound, (2) the study was conducted solely on human fetuses, and (3) the study contained information about genetic testing results or prognosis information. Cohort, retrospective, prospective, and longitudinal articles were included. Case reports, conference abstracts, comments and editorials were excluded. Pediatric, postnatal, adult and autopsybased studies were excluded. Articles in which isolated VSD category were included but could not be separated from others were excluded. To avoid duplication, the article with the longest study period or largest sample size was selected for final analysis in articles with overlapping locations and periods.

## 3 Prevalence

A meta-analysis of 260 studies that incorporated global data concluded that the reported birth prevalence of CHD globally continues to increase. Among the total CHD, the three mild lesions including VSD (occurred in 3.1 per 1,000 birth), atrial septal defect (ASD) and patent ductus arteriosus (PDA) together explained 93.4% of the increase prevalence (Liu et al., 2019). Although there are significant regional discrepancies in the incidence of VSD, this trend were also observed in the studies of Sweden and China, with a VSD birth prevalence of 7 per 1,000 and 1.4 per 1,000 respectively (Zhao et al., 2020a; Giang et al., 2023). Considering impact of the detection rate, intervention and spontaneous closure of the defect, prenatal statistics may vary from postnatal data. In a recent large-scale screening project of Chinese population, the incidence of isolated single VSD was 1.7 per 1,000 among pregnancies between 18 and 22 weeks’ gestational age nationwide, significantly higher than 0.1 per 1,000 of non-isolated VSD (Chen et al., 2023). Another register-based study from Denmark retreved nationwide data from routine ultrasound screening over a five-year period and found an incidence rate of isolated VSD of 0.5 per 1,000 low-risk pregnancies (Vedel et al., 2021).

## 4 Genetic association

The causes and pathological mechanisms of VSD serve as core information for assessing fetal prognosis, managing pregnancy, and parental decision-making. Clinicians should assess risk factors and discuss testing options in prenatal genetic counseling. Karyotype analysis is the gold standard for chromosomal abnormalities and the first-line method for prenatal diagnosis. However, it is controversial at present about the association between isolated VSD and the risk of chromosomal abnormality, thus puzzles the pre-test clinical counseling (Gómez et al., 2014; Shen et al., 2014; Lee et al., 2016; Svirsky et al., 2019; Vedel et al., 2021). The existence of additional risk indicators beyond isolated VSD may be the primary reason for the inconsistent genetic detection rates, as is shown in Tables 1, 2. In addition, geographical and socio-economic factors also lead to the deviation of the incidence. On the basis of conventional karyotype analysis, chromosomal microarray analysis (CMA) and exome sequencing (ES) have become more common in the prenatal setting in recent years. It also brings challenges for clinicians to choose approproate prenatal testing methods.

**TABLE 1:** General characteristics of the studies in fetuses with isolated VSD.

References	Country	Study design	Study obsject	Study period (years)	Related risk criteria^a^	Fetuses diagnosed as isolated VSD	GA at diagnosis (weeks)
Svirsky et al. (2019)	Israel	Retrospective	Isolated muscular VSD	2013–2017	√ Low risk for trisomy 21 on their first trimester screen × Other abnormal finding during the anomaly scan	40	After 15
Vedel et al. (2021)	Denmark	Prospective	Isolated VSD	2014–2018	√ cFTS risk for trisomy 21 was lower than 1:300 and lower than 1:150 for trisomy 18 and trisomy 13 calculated using the FMF algorithm √ Singleton pregnancy × Other prenatally detected malformations recorded × Second-trimester soft markers recorded: echogenic bowel, short femur, echogenic intracardiac focus, choroid plexus cyst	153	18–21
Lee et al. (2016)	USA	Retrospective	Isolated VSD	2006–2012	× Major intracardiac or extracardiac anomalies × Soft marker for aneuploidy: thickened nuchal skin fold, hypoplastic or absent nasal bone, echogenic bowel, ventriculomegaly, echongenic cardiac focus, renal pyelectasis, choroid plexus cyst, tricuspid regurgitation, short humerus or short femur, and 2 vessel cord × Abnormal serum screening × Multiple gestations	112	20.9 ± 1.6
Cai et al. (2018)	China	Retrospective	VSD	2017–2018	× Other cardiac anomalies × Extracardiac structural anomalies × Sonographic soft markers	79	18–33
Fu et al. (2017)	China	Retrospective	VSD	2010–2015	√ VSD as the only cardiac defect √ Normal karyotypes × Other cardiac anomalies × Extracardiac structural anomalies × Sonographic soft markers: echogenic foci in the heart or bowel, thickened nuchal fold, absent nasal bone, single umbilical artery, and persistent right umbilical vein, choroid plexus cysts	73	17–35
Du et al. (2016)	China	Retrospective	VSD	2013–2014	√ Normal karyotypes × Other cardiac anomalies × Extracardiac structural anomalies × Sonographic soft markers	22	18–34
Maya et al. (2020)	Israel	Retrospective	VSD	2013-2017	× Soft markers × Major anomalies × Growth/AFI anomalies	568	NS
Bahtiyar et al. (2008)	USA	Prospective	Isolated muscular VSD	2005–2006	× Any other sonographic abnormality in the fetus during a second-trimester anatomic survey × VSD as part of complex CHDs (e.g., atrioventricular septal defect, tetralogy of Fallot, and tricuspid atresia)	16	20–34
Shen et al. (2014)	Israel	Retrospective	Isolated VSD	1995–2007	× Maternal diabetes × Teratogenic exposure × Previous children with CHD × Prenatal diagnosis of a major malformation × Positive screen for trisomy 21 (on nuchal translucency testing with or without first trimester biochemical screening or second trimester triple testing with a calculated risk for DS greater than 1:380) × Soft signs for aneuploidy	92	19–24
Wang et al. (2023c)	China	Retrospective	VSD	2012–2022	× Sonographic soft markers × Extracardiac structural anomalies × Other cardiac anomalies × Known infected fetuses × Twin or multiple pregnancies × Exposure to known teratogenic drugs	133	12–38
Raucher Sternfeld et al. (2022)	Israel	Retrospective	Isolated VSD	2012–2015	× Other additional findings during anomaly scan	86	22–28
Cheng et al. (2022)	China	Retrospective	Isolated VSD	2016–2020	× Other ultrasound structural malformation at the time of antenatal and postnatal	185	23–27
Gordin Kopylov et al. (2022)	Israel	Retrospective	isolated perimembranous VSD	2015–2021	√ Perimembranous VSD was the only abnormal fetal finding × Additional cardiac or extracardiac malformations	56	22–29
Erol et al. (2014)	Turkey	Retrospective	Isolated muscular VSD	2007–2012	√ A defect in the interventricular septum without other sonographic abnormalities × Complex CHDs × Non-cardiac malformation × Known chromosomal abnormalities	76	19–37
Qiao et al. (2021)	China	Retrospective	CHD	2018–2019	× Additional extracardiac structural lesion	102	NS
Gómez et al. (2014)	Spain	NS	Isolated VSD	2005–2011	× Other structural anomalies at the time of diagnosis, i.e., other CHD, vascular anomalies and/or non-cardiac malformations	270	17–41
Mosimann et al. (2014)	UK	Retrospective	VSD	2002–2011	× Increased nuchal translucency × Extracardiac anormalies × Additional cardiac abnormalities	34	NS
Gedikbaşı et al. (2010)	Turkey	Retrospective	VSD	2002–2007	× Extracardiac findings × Co-existing cardiac findings	18	NS
Li et al. (2016)	China	Longitudinal	Isolated VSD	2011–2013	× Other cardiac structural abnormalities	335	19–30
Cho et al. (2017)	South Korea	Retrospective	Isolated VSD	2010–2014	× VSD as a part of another congenital heart defect (atrioventricular septal defect, tetralogy of Fallot, tricuspid atresia, *etc.*)	228	mid-trimester
Chau et al. (2018)	USA	Retrospective	VSD	2012–2015	× Other cardiac anomalies	129	NS
Paladini et al. (2000)	Italy	Observation	Isolated VSD	1994–1998	× VSD as a component of other CHD (tetralogy of Fallot, tricuspid atresia, etc.) or associated with other CHD (VSD + coarctation)	68	17–39
Axt-Fliedner et al. (2006)	Germany	Retrospective	Isolated VSD	1996–2004	× VSD as a part of other CHD (atrioventricular septal defect, tetralogy of Fallot, tricuspid atresia, etc.)	146	13–39
Selhorst et al. (2024)	USA	Retrospective	Isolated VSD	2018–2023	√ VSD with or without major structural abnormalities × Multiple heart abnormalities × Multiple gestation pregnancy	125	27 ± 5
Lu et al. (2024)	China	NS	CHD	2017–2022	× Extracardiac ultrasound anomalies × Additional structural anomalies × Soft markers × Amniotic fluid volume abnormality	219	24.3 ± 2.9
Zhao et al. (2024)	China	Retrospective	VSD	2017–2022	√ Low risk of NIPT × No test of NIPT × High risk of NIPT × Presence of other cardiac and extracardiac abnormalities	45	NS

√ included criteria,× excluded critieria, AFI, amniotic fluid index; cFTS, combined first-trimester screening; CHD, congenital heart disease; CMA, chromosomal microarray analysis; CNV, copy number variation; DS, down syndrome; ES, exome sequencing, GA gestational age; NIPT, non-invasive prenatal testing; NS, not stated; QF-PCR, quantitative fluorescence PCR.

^a^ Nine studies proposed definitions for the term “isolated” or “isolated VSD”, 11 studies established inclusion or exclusion criteria, and risk indicators related were extracted from full text of the remaining 6 articles.

**TABLE 2:** Detection rate of chromosomal aberration, CNV and SV in fetuses with isolated VSD.

References	Genetic testing	Total number of testing	Positive genetic diagnosis n(%)	Chromosomal aberrations n(%)	CNV n(%)	SV n(%)
Lee et al. (2016)	Karyotype	112	2 (1.79)	2 (1.79)		
Fu et al. (2017)	CMA	73	4 (5.48)		4 (5.48)	
Du et al. (2016)	CMA	22	1 (4.55)		1 (4.55)	
Maya et al. (2020)	CMA	568	8 (1.41)	1 (0.18)	7 (1.23)	
Cai et al. (2018)	Karyotype, CMA	79	2 (2.53)	1 (1.27)	1 (1.27)	
Svirsky et al. (2019)	Karyotype, CMA	30	2 (6.67)	1 (3.33)	1 (3.33)	
Vedel et al. (2021)	Karyotype, CMA	76	1 (1.32)	0 (0.00)	1 (1.32)	
Bahtiyar et al. (2008)	Karyotype	16	1 (6.25)	1 (6.25)		
Shen et al. (2014)	Karyotype	92	1 (1.09)	1 (1.09)		
Wang et al. (2023c)	QF-PCR, CMA, ES	133	7 (5.26)	2 (1.50)	4 (3.01)	1 (8.33, in 12 cases)
Erol et al. (2014)	Karyotype	18	0 (0.00)	0 (0.00)		
Gordin Kopylov et al. (2022)	CMA	30	0 (0.00)		0 (0.00)	
Raucher Sternfeld et al. (2022)	Karyotype, CMA	23	0 (0.00)	0 (0.00)	0 (0.00)	
Cheng et al. (2022)	QF-PCR, karyotype, CMA	170	9 (5.29)	2 (1.18)	7 (4.12)	
Qiao et al. (2021)	CMA, ES	102	23 (22.55)	10 (9.80)	7 (6.86)	6 (5.88)
Gómez et al. (2014)	Karyotype, FISH	248	3 (1.21)	3 (1.21)		
Mosimann et al. (2014)	Karyotype	33	0 (0.00)	0 (0.00)		
Gedikbaşı et al. (2010)	Karyotype	18	1 (5.56)	1 (5.56)		
Cho et al. (2017)	Karyotype	37	4 (10.81)	4 (10.81)		
Chau et al. (2018)	Karyotype	129	9 (6.98)	9 (6.98)		
Li et al. (2016)	Karyotype	80	9 (11.25)	9 (11.25)		
Paladini et al. (2000)	Karyotype	45	12 (26.67)	12 (26.67)		
Axt-Fliedner et al. (2006)	Karyotype	76	25 (32.89)	25 (32.89)		
Selhorst et al. (2024)	Karyotype, CMA	43	14 (32.56)	9 (20.93)	3 (6.98)	
Lu et al. (2024)	Karyotype, CMA	219	14 (6.39)	5 (2.28)	9 (4.11)	
Zhao et al. (2024)	Karyotype, CNV-seq	45	0 (0.00)	0 (0.00)	0 (0.00)	

CMA, chromosomal microarray analysis; CNV, copy number variant; ES, exome sequencing; FISH, fluorescence in situ hybridization; QF-PCR, quantitative fluorescence polymerase chain reaction; SV, sequence variant.

### 4.1 Chromosomal abnormality in isolated VSD

In early years, prenatal sonographic diagnosis of VSD was difficult. Large VSD was usually detected by prenatal ultrasound with cardiac and extracardiac abnormalities, and karyotype analysis was the most effective available detection method at that time. In the year of 2000 and 2006, Paladini et al. and Axt-fliedner et al. recruited fetal VSDs without associated cardiac defect and obtained high detection yields 26.67% and 32.89% of chromosomal abberation by karyotype analysis (Paladini et al., 2000; Axt-Fliedner et al., 2006). Trisomy 18 was the most common chromosomal aberration in fetal VSD without associated cardiac defect, followed by trisomy 21 and trisomy 13. Paladini et al. also reported a strong correlation between trisomy 21 and inlet VSD, as well as trisomy 18 and malalignment VSD (Paladini et al., 2000). Till the era of first-trimester screening, aneuploid was less prevalent in the cohort of isolated VSD without associated cardiac abnormalities. Li et al. from China performed karyotyping in 80 cases and identified 9 chromosomal anomalies, including 8 aneuploids and 1 balanced translocation (Li et al., 2016). Another study in East Asian population reported deleterious derivative chromosomes but no aneuploid in 4 fetuses (Cho et al., 2017). However, in these studies, the presence of extracardiac abnormalities has raised doubts about the association between chromosomal aberration and isolated VSD.

Some researchers concerned about the risk of fetal genetic abnormalities increased by isolated VSD itself and excluded VSD with extracardiac abnormalities from the prenatal cohort. During this period, sequential prenatal diagnostic method of cytogenetics and molecular genetics was widely adopted. Some authors believe that ultrasound soft markers woud not influence the postnatal cardiac management of VSD. Cheng et al. analyzed 170 invasive samples combining quantitative fluorescent polymerase chain reaction (QF-PCR), karyotyping analysis and CMA. Two aneuploids and seven copy number variations (CNVs) were identified, with detection rate of 1.18% for chromosomal aberration and 4.12% for CNV(Cheng et al., 2022). Interestingly, all of the positive variants were found in perimembranous VSD (Supplementary Table S1) (Cheng et al., 2022). Another large-scale study by Gómez et al. perfomed karyotyping and fluorescence in situ hybridization (FISH) in 119 amniotic fluid samples and clinically assessed karyotype postnatally in 129 cases, and obtained a total diagnostic yield of 3.13% in perimembranous VSD and 0.93% in muscular VSD. Gómez et al. concluded that perimembranous VSD was associated with a higher risk of chromosomal anomalies than muscular VSD and muscular VSD had a similar risk to those of normal pregnancies (Gómez et al., 2014). Qiao et al. also reported a total 16.66% detection rate of 17 aneuploides and CNVs in 102 isolated VSDs by CMA (Qiao et al., 2021).

Other researchers set rigorous inclusion criteria. In the cohort studies of isolated VSD without cardiac, extracardiac abnormalities or ultrasound soft markers, Fu et al. and Du et al. reported CMA diagnostic rates of 5.48% and 4.55% on the basis of normal karyotype, respectively (Du et al., 2016; Fu et al., 2017). These results also demonstrated the value of CMA in the prenatal diagnosis of isolated VSD (Du et al., 2016; Fu et al., 2017). However, such conclusion was questioned by Maya et al. (2020). In Israel, routinely reported CMA analysis supported by the Ministry of Health is performed for sonographic defect, allowing for a comparison among additional risk factors. In a cohort of 691 cases, 8 abnormal CMA results were found in 568 isolated VSD (1.4%), compared with 1 in 20 (5%) VSD with soft markers, 15 in 82 (18.3%) VSD with additional major anomalies and 2 in 21 (9.5%) VSD with growth or amniotic fluid index anomalies. Maya et al. suggested that the rate of abnormal CMA findings in isolated VSD was not different from pregnancies with normal ultrasound (Maya et al., 2020).

### 4.2 Sequence variant in isolated VSD

Chinese researchers have also made some attempts on the prenatal application of ES in isolated VSD. Qiao et al. conducted ES over CMA for fetuses with isolated VSD, resulted in an additional 5.6% diagnostic yield of sequence variant (SV). Among the 6 cases with SVs, 3 fetuses without additional abnormalities were detected to have genetic syndromes (Qiao et al., 2021). Wang et al. reported a likely pathogenic variation from 12 isolated VSD samples detected by ES (Wang et al., 2023c).

## 5 Prenatal genetic testing

### 5.1 Karyotyping analysis

In the overall prenatal diagnostic environment, karyotype analysis with high cost-effective ratio is often the first-line and irreplaceable choice for detection. For fetuses with VSD accompanied by other structural abnormalities, karyotype analysis is an appropriate option. For other clinical situations, it seems that additional indicators should be more considered in the risk assessment of chromosome abnormality, since isolated VSD itself does not increase or only slightly increases the risk of aneuploid. As a minor form of CHD, isolated VSD appears to be more associated with small genomic deletion and insertion, and monogenic disorders that have a insidious onset and non-fatal phenotype. However, A phenotypic shift has been reported in infants birth with Down syndrome from complex CHD to isolated VSD or ASD in recent years (Bergström et al., 2016). Chau et al. reported a trisomy 18 in isolated VSD fetus without any extracardiac defect or complications, diagnosed in the gestational age of 17^+5^ weeks (Chau et al., 2018). With the development of maternal-fetal medicine and improvement of prenatal care, it is possible that fetal phenotypes in the future may tend to be milder, and in this case, the proportion of chromosomal abnormalities may increase accordingly.

### 5.2 Chromosomal microarray analysis

CMA has been already confirmed as valuable tool and recommended to be used as the first-line genetic diagnostic testing for investigating the causes of fetal CHD (Thienpont et al., 2007; Southard et al., 2012; Mastromoro et al., 2022). Although authors disputed about the application of CMA in isolated VSD, CMA seems to be more efficient than karyotyping. Compared with karyotype analysis, CMA has high resolution and short turnaround, and was capable of identifying both chromosomal aneuploids and CNVs prenatally (Sukenik-Halevy et al., 2016; Zhu et al., 2016).

The deletion of 22q11.2 was the most common CNV identified in isolated VSD, while other CNVs occur in a sporadic form, such as 1q21.1 duplication and 16p11.2 duplication (Supplementary Table S2). With the features of reduced penetrance and pleiotropic effects, these CNVs in the genomic hotspots were reported to be enriched in CHD cohorts and affect dosage-sensitive genes that were required for cardiac development (Ehrlich and Prakash, 2022). Cheng et al. recommended CMA for fetal isolated VSD especially perimembranous subtype (Cheng et al., 2022). Besides, CMA also have clinical efficacy in exploring the etiology and pathogenesis of isolated VSD. Fu et al. identified potential candidate genes of VSD including *FAT1* and *ULK1* through prenatal testing by CMA (Fu et al., 2017). In some centers, array analysis has become a standard procedure for prenatal genetic analysis, and it is commonly preceded by rapid aneuploidy detection to exclude common aneuploidies, which can be an effective alternative to cytogenetics.

### 5.3 Exome sequencing

In recent years, ES has becoming a robust tool for prenatal diagnositic applications of CHD (Jin et al., 2017; Lord et al., 2019; Petrovski et al., 2019). Monogenic defects are known causitive factors for isolated VSD in the young (Pierpont et al., 2007). Pedigree studies have identified critical genes encoding important constituents of signaling pathways involved in controlling heart development. For these reasons, ES was also utilized in isolated VSD prenatally. In the two existing studies, desirable diagnostic increments were obtained by the application of ES (Qiao et al., 2021; Wang et al., 2023c). In addition, deleterious SVs in isolated VSD fetuses have been described in several CHD cohort studies (Fu et al., 2018; Li et al., 2020; Yi et al., 2023).

The interactions of multiple cardiac genes and their activation by up stream inductive signals maintain the cardiac phenotype (Chaithra et al., 2022). To date, researchers have mapped three gene loci (*GATA4*, OMIM#600576; *CITED2*, OMIM#602937; *NKX2.5*, OMIM#600584) related to the phenotype of VSD using sequencing technology (Table 3) (Sperling et al., 2005; Zhang et al., 2008; Peng et al., 2010). These gene variants lead to varying degrees of familial VSD with or without other cardiac abnormalities, such as ASD and pulmonary hypertension (Sperling et al., 2005; Chen et al., 2010; Wang et al., 2011a; Wang et al., 2011b; Yang et al., 2012). In addition, several genetic syndromes with varying severity and phenotypic heterogeneity, including Noonan syndrome, Kabuki syndrome, CHARGE syndrome and Holt-Oram syndrome, have been reported in fetal isolated VSD (Fu et al., 2018; Li et al., 2020; Qiao et al., 2021; Wang et al., 2023c; Yi et al., 2023). Due to the unintuitive physical characteristics of prenatal ultrasound and late onset phenotype of some syndromes, especially those with neurodevelpmental and psychiatric characteristics, isolated symptoms may be the only indication for those fetuses (Kabra and Gulati, 2003; Wang et al., 2020b).

**TABLE 3:** Summary of genes involved in fetal isolated VSD.

Gene	MIM number	Location	Phenotype	Inheritance	Protain	Function
*GATA4*	600,576	8p23.1	- Ventricular septal defect 1	AD	Gata-binding protein 4	One of the earliest transcription factors expressed in cardiac precursor cells; initiated ectopic cardiac gene expression(Välimäki et al., 2017)
			- Atrial septal defect 2	AD		
			- Atrioventricular septal defect 4	AD		
			- Tetralogy of Fallot	AD		
			- ?Testicular anomalies with or without congenital heart disease	AD		
*CITED2*	602,937	6q24.1	- Ventricular septal defect 2	AD	Cbp/P300 interacting transactivator with Glu/Asp rich carboxy-terminal domain 2	A cAMP-responsive element-binding protein (CBP)/p300-transactivator, function as an important transcriptional modulator(Su et al., 2016)
			- Atrial septal defect 8	AD		
*NKX2.5*	600,584	5q35.1	- Ventricular septal defect 3	AD	NK2 homebox 5	The earliest transcription factor expressed in all vertebrate cardiogenesis; involved in the whole process of heart development including cardiac precursor cell differentiation, cardiac cyclization, atrial compartmentalization, atrioventricular outflow tract and conduction system formation(Wang et al., 2020a)
			- Atrial septal defect 7, with or without AV conduction defects	AD		
			- Conotruncal heart malformations, variable			
			- Hypoplastic left heart syndrome 2	AD		
			- Hypothyroidism, congenital nongoitrous, 5	AD		
			- Tetralogy of Fallot	AD		
*TBX20*	606,061	7p14.2	- Atrial septal defect 4		T-box transcription factor 20	A transcriptional activator and repressor required for cardiac development(Perrot and Rickert-Sperling, 2024)
*TBX5*	601,620	12q24.21	- Holt-Oram syndrome	AD	T-box transcription factor 5	Participate in the differentiation of myocardial cells and atrioventricular cavities in the early stage of cardiac development; participate in the development of conduction system; maintain the function of mature myocardial cells in the later stage(Smemo et al., 2012)
*CHD7*	608,892	8q12.2	- CHARGE syndrome	AD	Chromodomain helicase DNA-binding protein 7	An ATP-dependent chromatin modifier; expression in the pharyngeal surface ectoderm and participate in formation of the great vessels; required for atrioventricular cushion development and septation of the outflow tract in the cardiogenic mesoderm; may act in concert with transcription factors such as TBX1 and SMADs to regulate genes such as p53 and the cardiac transcription factor NKX2.5(Meisner and Martin, 2020)
			- Hypogonadotropic hypogonadism 5 with or without anosmia	AD		
*PTPN11*	176,876	12q24.13	- Noonan syndrome 1	AD	Tyrosine-protein phosphatase non-receptor type 11	Acts downstream of various receptor and cytoplasmic protein tyrosine kinases to participate in the signal transduction from the cell surface to the nucleus(Pannone et al., 2017)
			- LEOPARD syndrome 1	AD		
			- Leukemia, juvenile myelomonocytic, somatic			
			- Metachondromatosis	AD		
*ANKRD11*	611,192	16q24.3	- KBG syndrom	AD	Ankyrin repeat domain-containing protein 11	A crucial chromatin co-regulator; control histone acetylation and gene expression by recruiting chromatin remodelers upon interaction with specific transcriptional repressors or activators(Gallagher et al., 2015)
*SON*	182,465	21q22.11	- ZTTK syndrome	AD	SON DNA-binding protein	RNA-binding protein; act as a mRNA splicing cofactor by promoting efficient splicing of transcripts that possess weak splice sites(Kim et al., 2016)
*SOS1*	182,530	2p22.1	- Noonan syndrome 4	AD	Son of sevenless homolog 1	A RAS-specific guanine nucleotide exchange factor; catalyzes the activation of the RAS-MAPK pathway(Baban et al., 2019)
			- ?Fibromatosis, gingival, 1	AD		
*KMT2D*	602,113	12q13.12	- Kabuki syndrome 1	AD	Histone-lysine N-methyltransferase 2D	H3K4me1 methyltransferase; critical for enhancer activation, cell differentiation and development(Xie et al., 2023)
			- Branchial arch abnormalities, choanal atresia, athelia, hearing loss, and hypothyroidism syndrome	AD		
*KRAS*	190,070	12p12.1	- Noonan syndrome 3	AD	KRAS Protooncogene GTPase	Ras proteins bind GDP/GTP and possess intrinsic GTPase activity(Gremer et al., 2011); Play an important role in the regulation of cell proliferation(Zimmermann et al., 2013)
			- Cardiofaciocutaneous syndrome 2	AD		
			- RAS-associated autoimmune leukoproliferative disorder	AD		
			- Arteriovenous malformation of the brain, somatic			
			- Bladder cancer, somatic			
			- Breast cancer, somatic			
			- Gastric cancer, somatic			
			- Leukemia, acute myeloid, somatic			
			- Lung cancer, somatic			
			- Oculoectodermal syndrome, somatic			
			- Pancreatic carcinoma, somatic			
			- Schimmelpenning-Feuerstein-Mims syndrome, somatic mosaic			

As concluded by Wang et al., ES can be reccommended for fetuses with VSD without chromosome abnormalities and pathogenic CNV (Wang et al., 2023c). Objectively, ES may have the potential to replace CMA and as a first-line diagnostic tool. Methods for calling CNV from ES data have been widely developed and in the process of clinical optimization, which expand the clinical practice by ES (D'Aurizio et al., 2016; Zhao et al., 2020b; Testard et al., 2022; Babadi et al., 2023). As the decline in costs and accumulation of data, it was undisputed that ES would be applied extensively in fetal isolated VSD in the following years.

### 5.4 Prenatal genetic screening

Lee et al. have attempted to investigate the impact of abnormal maternal serum screening on the detection rate of chromosomal abnormalities in isolated VSD (Lee et al., 2016). Among the two isolated VSD with trisomy 21, one has a high risk of serum screening and the other has not reveived the test (Lee et al., 2016). A few studies have also involved the contribution of non-invasive prenatal testing (NIPT) in prenatal finding of isolated VSD. In a single-center cohort study, 9 cases with high risk of chromosomal abnormalities were identified by NIPT from a total of 125 fetuses with isolated VSD (Selhorst et al., 2024). Despite the lack of detailed corresponding genetic results, their results indicated the NIPT has promoted the early prevention of chromosome-related VSD (Selhorst et al., 2024). Zhao et al. discussed that fetuses with isolated VSD and low-risk of NIPT might not need invasive prenatal diagnosis because no genetic variant was found in 45 NIPT low-risk isolated VSD fetuses by karyotype and CNV-seq in their study (Zhao et al., 2024). However, a case of neonatal death due to metabolic disease has not been reasonably explained which might mean the potential missed diagnosis of monogenic disease. The exploration of NIPT for monogenic diseases is under way. Due to the variable severity and phenotypic heterogeneity, it is possible to discuss in the future whether VSD-related genes can be included in the NIPT for monogenic diseases.

## 6 Spontaneously closure, pregnancy outcome and long-term follow-up

Most of the isolated VSDs detected by fetal ultrasound could close spontaneously during pregnancy and infancy (Table 4). Size, site and maternal age are prognostic factors for the natural closure of the defect (Paladini et al., 2000; Cho et al., 2017; Cheng et al., 2022; Gordin Kopylov et al., 2022). The spontaneous closure mechanisms of muscular and perimembranous isolated VSD were different and have been well elaborated, as reviewed by Miyake (Miyake, 2020). For fetuses, authors regard muscular isolated VSD as benign finding, while perimembranous VSD tends to have larger size, lower closure rates, and more likely require treatment by medications, special care or surgery to thrive (Gómez et al., 2014; Li et al., 2016; Cho et al., 2017; Svirsky et al., 2019; Cheng et al., 2022; Raucher Sternfeld et al., 2022; Zhao et al., 2024). Li et al. also found a higher spontaneous closure rate in male than female (Li et al., 2016).

**TABLE 4:** Rate of spontaneous closure of the isolated VSD.

References	Latest follow-up age	Number of follow-up	Intrauterine closure n(%)	Closure before age of 12 months n(%)	Total closure n(%)	No closure, surgery or death n(%)
Vedel et al. (2021)	Birth	153	71 (46.41)		71 (46.41)	82 (53.59)
Lee et al. (2016)	Birth	112	73 (65.18)		73 (65.18)	39 (34.82)
Fu et al. (2017)	12 months	69		42 (60.87)	42 (60.87)	27 (39.13)
Svirsky et al. (2019)	3–48 months	26	13 (50.00)		21 (80.77)	5 (19.23)
Erol et al. (2014)	12 months	45	3 (6.67)	33 (73.33)	33 (73.33)	12 (26.67)
Gordin Kopylov et al. (2022)	12 months	56	25 (44.64)	42 (75.00)	42 (75.00)	14 (25.00)
Gómez et al. (2014)	12 months	213	13 (6.10)	164 (77.00)	164 (77.00)	49 (23.00)
Raucher Sternfeld et al. (2022)	24 months	75	34 (45.33)	58 (77.33)	64 (85.33)	11 (14.67)
Cheng et al. (2022)	24 months	168	48 (28.57)	79 (47.02)	101 (60.12)	67 (39.88)
Chau et al. (2018)	Birth	129	120 (93.02)		120 (93.02)	9 (6.98)
Paladini et al. (2000)	12 months	68	13 (19.12)	19 (27.94)	19 (27.94)	49 (72.06)
Axt-Fliedner et al. (2006)	12 months	139	37 (26.62)	87 (62.59)	87 (62.59)	52 (37.41)
Cho et al. (2017)	3 days to 60 months	149	64 (42.95)		99 (66.44)	50 (33.56)
Li et al. (2016)	NS	257	49 (19.07)		110 (42.80)	147 (57.20)
Zhao et al. (2024)	At least 6 months	37		7 (18.92)	7 (18.92)	30 (81.08)

NS, not stated.

Remarkably, as evidenced by the findings of Selhorst et al., genetic variations manifested with a heightened prevalence in infants whose VSD underwent spontaneous closure in utero, compared to infants who exhibited persistent VSD (Selhorst et al., 2024). Genetic abnormalities and extracardiac abnormalities are the main reasons for termination of pregnancy (TOP) and adverse pregnancy outcomes (Table 5). Most of the other isolated VSD fetuses have a positive pregnancy outcome and no need for surgery. A few referral to pediatric surgical department usually have low operative mortality and good prognosis when treated in a timely manner (Nurmi et al., 2022). However, it has also been reported that unrepaired and surgically closed isolated VSD affected the long-term survival of patients and was prone to late complications (Eckerström et al., 2023). The recent advances in minimally invasive treatment options including periventricular approach and transcatheter techniques have improved patient outcomes, yet at the expense of higher residual rates (Adan et al., 2021). Right ventricular function and exercise capacity were found impaired in VSD patients, and post-surgical outcome in these patients may be less benign than presently assumed (Nederend et al., 2018; Eckerström et al., 2020; Adan et al., 2021).

**TABLE 5:** Outcome of fetuses with isolated VSD.

References	Number of follow-up	TOP n(%)	IUD n(%)	NND n(%)	ID n(%)	Survived n(%)	Surgery n(%)
Svirsky et al. (2019)	26	0 (0.00)	0 (0.00)	0 (0.00)	0 (0.00)	26 (100.00)	0 (0.00)
Cai et al. (2018)	79	2 (2.53)	NS	NS	NS	NS	NS
Fu et al. (2017)	69	4 (5.80)	0 (0.00)	0 (0.00)	0 (0.00)	65 (94.20)	11 (15.94)
Vedel et al. (2021)	153	4 (2.61)		NS	NS	NS	NS
Shen et al. (2014)	92	0 (0.00)	0 (0.00)	0 (0.00)	NS	92 (100.00)	NS
Gordin Kopylov et al. (2022)	56	0 (0.00)	0 (0.00)	1 (1.79)	0 (0.00)	55 (98.21)	3 (5.36)
Erol et al. (2014)	45	0 (0.00)	0 (0.00)	0 (0.00)	1 (2.22)	44 (97.78)	NS
Raucher Sternfeld et al. (2022)	75	0 (0.00)	0 (0.00)	0 (0.00)	0 (0.00)	75 (100.00)	3 (4.00)
Cheng et al. (2022)	168	7 (4.17)	0 (0.00)	0 (0.00)	0 (0.00)	161 (95.83)	33 (19.64)
Gómez et al. (2014)	213	1 (0.47)	0 (0.00)	0 (0.00)	1 (0.47)	211 (99.06)	7 (3.29)
Gedikbaşı et al. (2010)	18	1 (5.56)	1 (5.56)	0 (0.00)	0 (0.00)	16 (88.89)	NS
Cho et al. (2017)	149	1 (0.67)	2 (1.34)	0 (0.00)	NS	146 (97.99)	4 (2.68)
Li et al. (2016)	257	44 (17.12)	0 (0.00)	8 (3.11)		205 (79.77)	19 (7.39)
Paladini et al. (2000)	68	28 (41.18)	2 (2.94)	12 (17.65)	0 (0.00)	26 (38.24)	NS
Axt-Fliedner et al. (2006)	139	23 (16.55)	0 (0.00)	1 (0.72)	0 (0.00)	113 (81.29)	NS
Zhao et al. (2024)	45	0 (0.00)	0 (0.00)	1 (2.22)	0 (0.00)	44 (97.78)	7 (15.56)

ID, infant death; IUD, intrauterine death; NND, neonatal death; NS, not stated; TOP, termination of pregnancy.

Several individuals with isolated VSD have developed extra abnormalities during late pregnancy to infancy, such as esophageal atresia, coarctation of the aorta and pulmonary stenosis, which suggested that clinicians should monitor the associated abnormalities in the subsequent period (Bensemlali et al., 2017; Fu et al., 2017; Cheng et al., 2022; Raucher Sternfeld et al., 2022). A specific link between VSD and central nervous system anomalies has been described by Huang et al., but the detailed mechanisms underlying the breadth of co-occurring anomalies have yet to be delineated (Huang et al., 2023). Due to the limitation of follow-up time and retrospective study exclusion criteria, the results of this aspect have not been well-described in the prenatal cohort. Noncardiac anomalies are crucial for perioperative management and etiology study, and can also increase the risk of postoperative complications, such as respiratory complications with heterotaxy (Swisher et al., 2011; Harden et al., 2014). The complete record including mild and late-onset phenotypes, especially neuropsychiatric symptoms by long-term follow-up, was important for the forward evaluation of prognosis.

## 7 Prenatal counseling

The term “isolated VSD” was originally defined anatomically limited to the heart, referring to VSD without other cardiac defect (e.g. ASD) or as part of complex CHD (e.g. tetralogy of Fallot). However, in the prenatal setting, clinicians need to comprehensively evaluate the fetal prognosis based on the overall pregnancy to assess the risk. The risk brought by each indicator and the combined risk of multiple indicators should be deeply concerned.

The associated genetic abnormalities strongly influences the parent’s decision to choose postnatal compassionate care or TOP. CNV and SV with phenotypic heterogeneity and incomplete penetrance were more likely to cause fetal isolated VSD than chromosomal aberration. Clinicians should be aware of the benefits and implications of the responsible use of genomics. At the same time, issues raised by the prenatal detection of possible complex disorders from mild phenotypes should be taken into account:

- Balance of information disclosure. Clinicians should provide sufficient information to help parents understand the risk of disease, while ensuring that they do not unduly worry them, that is, balancing the completeness of the information with the parents’ ability to cope.
- Variants of unknown significance (VOUS). Clinicians should fully communicate with the parents to ensure that they understand the significance and limitations of the test results, while avoiding over-interpretation or misleading. It is necessary to set appropriate informed consent before testing. Researches are sometimes needed to verify the clinical significance, and it must follow the principles of medical ethics, ensuring it conforms to ethical standard and the informed consent from parents is obtained.
- Parental decision-making pressure. Clinicians should ensure that parents fully understand the genetic nature and associated risks of isolated VSD, so that they can make informed decisions. However, this may also place excessive decision-making pressure on parents, making it difficult to make a choice.
- Rights of the fetus. When considering the wishes and needs of the parents, it is also necessary to fully protect the rights of the fetuses, including their right to health and future autonomous decision-making.
- Overdiagnosis and overintervention. In some cases, parents may request excessive diagnosis or intervention, which may pose unnecessary risks to the fetus. Clinicians should carefully assess these risks and ensure that parents’ decisions are based on sufficient medical evidence.
- Resource allocation. As genetic counseling and testing may require significant medical resources and funding, clinicians need to ensure that these resources are allocated fairly and reasonably, so that all those need it can receive necessary services.
- Legal and ethical framework. Clinicians should understand and comply with relevant laws and ethical frameworks to ensure that their actions meet legal requirements and ethical standards. This includes respecting the rights of parents, protecting the rights of the fetus, and ensuring the confidentiality of information.

## 8 Future prospective

Advances in genomic technology reshape the practice of prenatal counseling in isolated VSD. Precision genome-wide detection will play a role in prenatal diagnosis, and further reveal the genetic mechanism of isolated VSD in the future. Seeking biomarkers for the prediction of fetal VSD is another research orientation in the assessment of health and disease. VSD-ralated specific IncRNAs and microRNAs have been authenticated in maternal serum and expected to serve as prenatal VSD diagnostic markers (Jin et al., 2021; Yang et al., 2022; Wang et al., 2023a; Wang et al., 2023b). Another differentially expressed protein CFHR4 was also identified as a promising biomarker (He et al., 2022). More omics analysis are expected to accurately predict the risk of VSD in fetuses, and thus providing basis for early intervention and treatment.
