# Supplementary material for: Prenatal finding of isolated ventricular septal defect: genetic association, outcomes and counseling
Source: Front Genet. 2024 Oct 2;15:1447216. doi: 10.3389/fgene.2024.1447216 (PMC11479991; doi:10.3389/fgene.2024.1447216)
Supplement: Supplementary file 1 [file Table1.docx]

Supplementary table 1. Incidence of chromosomal abnormalities in different subtypes of isolated VSD

| References | Genetic testing | Number of detected | Number of clinically significant genetic results  n(%) |
| --- | --- | --- | --- |
| Isolated muscular VSD | | | |
| (Svirsky et al., 2019) | Karyotype, CMA | 30 | 2 (6.67) |
| (Cheng et al., 2022) | CMA | 90 | 0 (0.00) |
| (Erol et al., 2014) | Karyotype | 18 | 0 (0.00) |
| (Gómez et al., 2014) | Karyotype, FISH | 216 | 2 (0.93) |
| (Mosimann et al., 2014) | Karyotype | 24 | 0 (0.00) |
| (Gedikbaşı et al., 2010) | Karyotype | 6 | 0 (0.00) |
| (Paladini et al., 2000) | Karyotype | 7 | 2 (28.57) |
| (Axt-Fliedner et al., 2006) | Karyotype | 66 | 19 (28.79) |
| Isolated perimembranous VSD | | | |
| (Cheng et al., 2022) | CMA | 78 | 7 (8.97) |
| (Gordin Kopylov et al., 2022) | Karyotype | 30 | 0 (0.00) |
| (Gómez et al., 2014) | Karyotype, FISH | 32 | 1 (3.13) |
| (Mosimann et al., 2014) | Karyotype | 9 | 0 (0.00) |
| (Gedikbaşı et al., 2010) | Karyotype | 12 | 1 (8.33) |
| (Paladini et al., 2000) | Karyotype | 35 | 12 (34.29) |
| (Axt-Fliedner et al., 2006) | Karyotype | 10 | 6 (60.00) |

*CMA* chromosomal microarray analysis, *FISH* fluorescence in situ hybridization, *VSD* ventricular septal defect

Supplementary table 2. Details of pathogenic/likely pathogenic CNVs in fetuses with isolated VSD

| References | GA at diagnosis  of VSD | Size  (mm) | Other findings | Type of VSD | CMA result/location | Size of CNV  (Mb) | CNV type | Classification | OMIM or corresponding disorder | Parental study | Outcome |
| --- | --- | --- | --- | --- | --- | --- | --- | --- | --- | --- | --- |
| (Svirsky et al., 2019) | NS | NS | No | Muscular | Xp22.31 | 1.3 | Deletion (heterozygosis) | Pathogenic | Ichthyosis, X-linked | NS | Live birth |
| (Cheng et al., 2022) | 25+6 | 2 | Hypoplastic nasal bone | Perimembranous | arr[hg19]4p16.3p15.33(68345_14195870)×1 | 14.13 | Deletion | Pathogenic | Wolf-Hirschhorn syndrome | de novo | TOP |
| (Cheng et al., 2022) | 23+3 | 1.8 | Echogenic intracardiac focus | Perimembranous | arr[hg19]22q11.21q11.23(21465661_23810042)×1 | 2.34 | Deletion | Pathogenic | Chromosomal 22q11.2 deletion syndrome, distal | de novo | TOP |
| (Cheng et al., 2022) | 26+2 | 2.7 | PLSVC | Perimembranous | arr[hg19]11q24.2q25(126039017_134938470)×1 | 8.9 | Deletion | Pathogenic | Jacobsen syndrome | NS | TOP |
| (Cheng et al., 2022) | 28+4 | 3.5 | No | Perimembranous | arr[hg19] 22q11.21(18648866_21465662)×1 | 2.82 | Deletion | Pathogenic | 22q11.2 deletion syndrome | de novo | TOP |
| (Cheng et al., 2022) | 27+1 | 4.2 | No | Perimembranous | arr[hg19]1q21.1q21.2(146488131_147819294)×3 | 1.33 | Duplication | Pathogenic | Chromosomal 1q21.1 duplication syndrome | Paternally inherited | TOP |
| (Cheng et al., 2022) | 23+0 | 3.8 | Choroid plexus cysts | Perimembranous | arr[hg19] 16p13.11(15140210_16326223)×3 | 1.19 | Duplication | Pathogenic | 16p13.11 recurrent region (BP2-BP3)(includes MYH11) | de novo | TOP |
| (Cheng et al., 2022) | 24+4 | 4.5 | No | Perimembranous | arr[hg19] 22q11.21(18648855_21800471)×1 | 3.15 | Deletion | Pathogenic | 22q11.2 deletion syndrome | de novo | TOP |
| (Vedel et al., 2021) | NS | NS | No | NS | 8q23.1 | NS | Deletion | Pathogenic | CHD, congenital diaphragmatic hernia and developmental delay | NS | NS |
| (Cai et al., 2018) | NS | NS | No | NS | arr[hg19]3q29(195743957-197386180)×3 | 1.6 | Duplication | Pathogenic | Chromosomal 3q29 microduplication syndrome | de novo | TOP |
| (Fu et al., 2017) | NS | NS | No | NS | 22q11.21 (18636749_21800471) | 3.16 | Deletion | Pathogenic | 22q11.2 deletion syndrome | NS | NS |
| (Fu et al., 2017) | NS | NS | No | NS | Xp21.1 (31590547_31923772) | 0.33 | Deletion | Pathogenic | Duchenne/Becker muscular dystrophy | de novo | NS |
| (Fu et al., 2017) | NS | NS | No | NS | 4q35.1q35.2 (186148866_189539349) | 3.39 | Deletion | Pathogenic | Terminal 4q  deletion  syndrome | NS | Live birth; 1 year old at time of study; surgical repair at 1 month; no dysmorphism |
| (Fu et al., 2017) | NS | NS | No | NS | 9q34.3 (138308948_141018648) | 2.71 | Deletion | Pathogenic | 9q subtelomeric  deletion  syndrome | de novo | NS |
| (Du et al., 2016) | NS | NS | Broad thumbs and first toes, polydactylism, pulmonary fibrosis | NS | arr[hg19]10q22.3(81298315-81422268)×3; arr [hg19]16p13.3(3793680-3998261)×1 | 0.12; 0.2 | Duplication; deletion | Pathogenic; pathogenic | Rubinstein-Taybi syndrome | NS | Live birth |
| (Maya et al., 2020) | NS | NS | No | Large perimembranous | arr5q33.2q35.2(155606954-173563033)×1 | 17.96 | Deletion | Pathogenic | Ventricular septal defect 3; tetralogy of Fallot; hypothyroidism, congenital nongoitrous, 5; hypoplastic left heart syndrome 2; atrial septal defect 7 | NS | NS |
| (Maya et al., 2020) | NS | NS | No | NS | arr7q11.22(69356386-69564376)×1 | 0.21 | Deletion | Pathogenic | Autosomal dominant mental retardation type 26 | NS | NS |
| (Maya et al., 2020) | NS | NS | No | Small midmuscular | arr16p11.2(29428531-30190029)×3 | 0.76 | Duplication | Pathogenic | 16p11.2 duplication syndrome | NS | NS |
| (Maya et al., 2020) | NS | NS | No | NS | arr16p12.2(21789785-22422637)×1 | 0.63 | Deletion | Pathogenic | 16p12.2 deletion syndrome | Paternal | NS |
| (Maya et al., 2020) | NS | NS | No | NS | arr16p13.11(14892975-16527659)×3 | 1.63 | Duplication | Pathogenic | 16p13.11 recurrent region (BP2-BP3)(includes MYH11) | Maternal | NS |
| (Maya et al., 2020) | NS | NS | No | NS | arr22q11.21(18919477-21800471)×1 | 2.88 | Deletion | Pathogenic | 22q11.2 deletion syndrome | NS | NS |
| (Maya et al., 2020) | NS | NS | No | Small midmuscular | arr22q11.1q11.21(16888899-18649190)×3 | 1.76 | Duplication | Pathogenic | 22q11.21 recurrent (Cat eye syndrome) region (includes CECR2) | NS | NS |
| (Maya et al., 2020) | NS | NS | No | Midmuscular | arrXp22.33q28(1-155270560)×1-2 | 155 | Mosaic deletion | Pathogenic | Mosaic Turner syndrome | de novo | NS |
| (Qiao et al., 2021) | NS | NS | No | NS | arr22q11.21(18889490-21811991)×1 | 2.92 | Deletion | Pathogenic | 22q11.2 deletion syndrome | NS | NS |
| (Qiao et al., 2021) | NS | NS | No | NS | arr13q21.33q31.3(70947451-90541300)×1 | 19.59 | Deletion | Pathogenic | Partial monosomy 13q syndrome | NS | NS |
| (Qiao et al., 2021) | NS | NS | Echogenic bowel | NS | arr22q11.21(18631364-21927646)×1 | 3.3 | Deletion | Pathogenic | 22q11.2 deletion syndrome | NS | NS |
| (Qiao et al., 2021) | NS | NS | No | NS | arr12p13.33q11(173786-37869107)×4 | 37.7 | Duplication | Pathogenic | Pallister-Killian Syndrome | NS | NS |
| (Qiao et al., 2021) | NS | NS | Single umbilical artery | NS | arr5p15.33p13.3(113576-33717880)×1 | 33.6 | Deletion | Pathogenic | Cri du chat syndrome | NS | NS |
| (Qiao et al., 2021) | NS | NS | No | NS | arr16p11.2(28807417-29032280)×1 | 0.22 | Deletion | Pathogenic | 16p11.2 microdeletion syndrome | NS | NS |
| (Qiao et al., 2021) | NS | NS | No | NS | arr8p23.3p23.2(17818-5387967)×1 | 5.37 | Deletion | Pathogenic | Terminal 8p deletion syndrome | NS | NS |
| (Lu et al., 2024) | NS | NS | No | NS | arr2q13(111382573-113111856)×1 | 1.72 | Deletion | Likely pathogenic | 2q13 recurrent region (includes BCL2L11) | Maternal | Live birth |
| (Lu et al., 2024) | NS | NS | No | NS | arr4q32.1q35.2(156228522_190957460)×3 | 34.72 | Duplication | Pathogenic | Partial trisomy 4q syndrome | de novo | TOP |
| (Lu et al., 2024) | NS | NS | No | NS | arr6q16.3q21(104495018_112902562)×3 | 8.40 | Duplication | Likely pathogenic | Partial trisomy 16q | NS | TOP |
| (Lu et al., 2024) | NS | NS | No | NS | arr16p13.12p13.11(14770632_16458424)×1 | 1.68 | Deletion | Pathogenic | 16p13.11 recurrent region (BP2-BP3) (includes MYH11) | NS | Live birth |

*CHD* congenital heart disease, *CMA*, chromosomal microarray analysis, *CNV* copy number variation, *GA* gestational age, *NS* not stated, *PLSVC* persistent left superior vena cava, TOP termination of pregnancy, *VSD* ventricular septal defect
